# Supplementary material for: Learning curve and surgical outcome of robotic assisted colorectal surgery with ERAS program
Source: Sci Rep. 2022 Nov 29;12:20566. doi: 10.1038/s41598-022-24665-w (PMC9709162; doi:10.1038/s41598-022-24665-w)
Supplement: Supplementary file 1 — Supplementary Information 1. [file 41598_2022_24665_MOESM1_ESM.zip › LSC Raw data-Ñ[▒K/094/H_ñw╝╨░O▒Kñσ.pdf]

正式摘要(電子簽章：000000199988638)

W55 / W55 ☐英文標題(注意:無英文姓名-請補病人基本檔!) ☐【讀取】憑證

臺中榮民總醫院

出院病歷摘要

轉入號： 轉出號：

[redacted]

(8)流水編號	
(9)入院日期	109/09/23 (2020)
(10)轉科日期	
(11)出院日期	109/09/30 (2020) 住院天數計 7 日

診斷

- 入院 :1.Distal T-colon tumor with bowel obstruction
  , s/p Loop colostomy (Proximal T) on 2020/09/07
  2.asymptom gallbladded stone
- 出院 :1.Distal T-colon tumor with bowel obstruction
  , s/p Loop colostomy (Proximal T) on 2020/09/07
  , s/p lap RH 6+ closure of ostomy on 2020/09/24 , c/w ostomy wound infection 2.asymptom gallbladded stone , s/p LC on 2020/09/24

主訴

- for scheduled operation

病史

- This is a 56 years old female with the history of
  1.Uterine leiomyoma,large uterine myoma with central infarction and adenomyosis s/p aparoscopy assisted vaginal hysterectomy in 中榮H
  2. Right clavicle fracture s/p operation in 光田H

  According to the patient,she had refractory abdominal cramping pain for one week ago. She went to 光田H for help on 9/7, where abdominal CT showed bowel dilatation of small bowel to transverse colon.For second opinion,she was transferred to our ER.At our ER,loop colostomy was done.Dilated bowel was noted.Loop colostomy (Proximal T) was done on 2020/09/07. This time, she was admitted for Lap RH+LC.

體檢發現

-
  - General appearance :
    - easy looking , conscious clear , without cardiopulmonary distress
    - Vital signs自動帶入date 體溫(BT) 脈搏(P) 呼吸(R) 血壓(BP) 身高(BH) 體重(BW) BMI
      20200923 36.7℃ 92次/分 18次/分 128/89mmHg
    - Vital signs: B.P. 153/70 mmHg , P.R. 80 /min , R.R. 19 /min , B.T. 36 ℃
  - HEENT :
    - Eyes: no anemic conjunctiva, no icteric sclera, normal EOM
    - ENT: no otorrhea, no rhinorrhea, no erythematous mucosa, no injected tonsil, no deformity
  - Neck: supple, freely movable, trachea midline,no deformity, no engorged jugular vein , no palpable thyroid or lymph node enlargement
  - Chest:
    - Inspection: Grossly normal, symmetric and fully expansion, no accessory muscle used, no operation scar, no spider angiomata, no gynecomastia
    - Palpation: no palpable mass or nodule, no subcutaneous emphysema, no tenderness
    - Ausculation: clear breathing sound
  - Heart:
    - Percussion: normal heart size
    - Ausculation: regular heart beat, normal S1, S2, no gallop, no murmur
  - Abdomen:
    - Inspection: in
      - flat contour, symetrically, no scar, no stria, no dilated veins, no rashes
    - Ausculation: normoactive bowel sound, no abdominal bruits
    - palpation: soft, no muscle guarding or rigidity, no local tenderness, no rebounding pain, no palpable mass, no palpable pulsation
    - Percussion: no shifting dulllness, no hepatomegaly, no splenomagaly
  -

Peripheral pulsation :	CA	BA	RA	FA	PA	PTA	DPA
Right side :	++	++	++	++	++	++	++/+++
Left side :	++	++	++	++	++	++	++/+++

  - Genito-anus: no tenderness or palpable mass lesion or rectal shelf, normal external genitalia, normal anal tone, brown stool

- **CRS CONDITION**
    -
        - Lung

手術日期及方法

- 手術日期及方法20200924 Operative methods:
  1. Laparoscopic right hemicolectomy with side to side anastomosis, endo GIA 45-3.5mm*3, re-inforced with 3-0 silk lamber suture.
  2. Segmental resection of small intestine, with side-to-side anastomosis with endo GIA 45.3.5mm*2, re-inforced with 3-0 silk lamber suture.
  3. Closure of T loop colostomy
  4. Vessel ligation: ileo-colic and right branches of middle colic vessels.
  5. Cut margin: proximal 19cm(colon), 5cm(ileum), distal 5cm.
  6. Laparoscopic cholecystectomy

  20200924 Laparoscopic Cholecystectomy

住院治療經過

- After admission, operation was done on 9/24. The ostomy wound wet dressing was done. However, much pus was noted on 9/28. We opened the wound on 9/29 and keep BID wet dressing. We will keep wet dressinig and plan to closure the wound at next week OPD. Under stable condition, we arranged discharge for her.

合併症

一般檢查

- 累積報告：

DATE	TIME	ALB	TP	BIL.T	BIL.D	ALKP	AST	ALT	LDH	CHOL	TG	HDL-C	LDL	UA	NH3	AMY
1090907	1815					100		20	217							
1090910	0919	3.6		0.2	0.1		15	11								
1090923	1139	4.4		0.41	0.11	83	18	16	171							
1090923	1713		7.7							182	149		118	5.2		

DATE	TIME	WBC	RBC	HGB	HCT	MCV	PLT	BLST	NEUT	BAND	SEG	LYM	MON	EOS	BAS
1090907	1812	8580		8.7			555		72.3			19.9	7.5	0.1	0.2
1090910	0903	7780	4.00	7.2	25.1	62.8	434		68.3			22.5	7.7	1.4	0.1
1090923	1139	5230	4.89	10.5	34.6	70.8	449		50.9			36.1	5.9	6.5	0.6
1090928	0942	7590	4.12	9.2			292		59.9			23.6	7.8	8.4	0.3

DATE	TIME	NA	K	CL	CA	BUN	CREAT	Mg	P	CRP	ESR	PCT	LIPASE	LACTATE
1090907	1815	134	4.2				0.69			1.093				12.9
1090910	0919	136	4.6		8.4	8	0.65							
1090923	1139	142	3.9		9.3	8	0.73							
1090923	1713									0.359				
1090928	0936	138	3.8			8	0.65							

DATE	TIME	GLUCOSE	PAB	TRF	ZN	CU	KETONE	CK	CKMB	PT-P	PT-C	APTT-P	APTT-C	TRO-T
1090907	1812									10.8	10.3	27.0		
1090907	2122	93												
1090923	1139	105								10.4	10.3	26.4		

DATE	TIME	SP-GR	PH	PROT	GLUC	KETON	BILI	UROB	OB	NITR	LEU	RBC	WBC	EPCEL
1090907	1842	>1.040	6.5	1+	Negative	2+	Negative	0.2	Negative	Negative	Negative	0-2	2-5	2-4

特殊檢查

病理報告

其它

出院時情況

- 3.改門診治療

出院指示

藥名	劑量	劑量單位	用法	頻次	數量	數量單位	現況	天數	單位
CefaDROxil-500 cap	1	CAP	PO	Q12H	14	CAP	TH	7	天
Ultracet tab	1	TAB	PO	QID	28	TAB	TH	7	天
Loperamide cap 2mg	1	CAP	PO	QNPRN	3	CAP	TH	3	天

出院計劃

主治醫師_2062F 蔣鋒帆 / 住院醫師_2330E 楊捷儒

正式摘要(電子簽章：**000000199988638)**

W55 / W55

doctorRank:B
